# Characterization of Aromatase Binding Agents from the Dichloromethane Extract of *Corydalis yanhusuo* Using Ultrafiltration and Liquid Chromatography Tandem Mass Spectrometry

**DOI:** 10.3390/molecules15053556

**Published:** 2010-05-14

**Authors:** Jing Shi, Xiaoyu Zhang, Zhongjun Ma, Min Zhang, Fang Sun

**Affiliations:** 1 Department of Pharmacy, Zhejiang Medical College, No.481 Binwen Rd., Hangzhou 310053, China; E-Mail: shij136@hotmail.com (J.S.); 2 School of Pharmaceutical Sciences, Zhejiang University, Zijingang Campus, No.388 Yuhangtang Rd., Hangzhou 310058, China; E-Mail: zhangxy_zju@hotmail.com (X.Z.)

**Keywords:** *Corydalis yanhusuo*, alkaloids, aromatase, ultrafiltration LC-MS

## Abstract

Aromatase represents an important target for the treatment of hormone-dependent breast cancer. In the present study, nine alkaloids from the dichloromethane extract of *Corydalis yanhusuo* were identified by liquid chromatography tandem mass spectrometry (LC-MS/MS) and tested for their aromatase binding activities using an ultrafiltration LC-MS method by investigating the differences of peak areas of compounds before and after incubations with aromatase. It was demonstrated that the quaternary protoberberine alkaloids and the tertiary protoberberine alkaloids exhibited potent aromatase binding activities. The quaternary ammonium group and the methyl group at C-13 position of tertiary protoberberine alkaloids might be necessary for the activity. The findings should provide guidance for the discovery of potential aromatase inhibitors from natural products.

## 1. Introduction

Breast cancer is a worldwide health problem and remains one of the leading causes of death among women. Approximately one-third of all breast cancers are hormone (estrogen) dependent. Though a variety of different treatment options are currently available, the incidence and mortality of breast cancer remain extremely high up to the present. It was widely recognized that estrogens and estrogen receptors (ERs) play key roles in the development and progression of hormone-dependent breast cancer [1]. With regard to the mechanisms involved in above process, two general strategies have been developed for the prevention or therapy of hormone-dependent breast cancer [2]. The first strategy is to introduce anti-estrogen agents such as tamoxifen to inhibit the binding of estrogens to ERs [3]. The second approach is to use aromatase inhibitors (AIs) to decrease the circulating levels of estrogens by blocking the biosynthesis of estrogens from androgens [4].

Aromatase, encoded by the CYP19 gene, is expressed in various tissues including ovary, placenta, bone, brain, liver, muscle, subcutaneous fat and normal breast tissues [5]. It is a key enzyme involved in the estrogen biosynthesis by converting androstenedione to estrone, or testosterone to estradiol. Deprivation of estrogens by AIs has been proved one of the most effective endocrine treatment strategies of breast cancer in previous study [6,7]. Until now, it has been demonstrated that some synthetic AIs such as anastrozole, letrozole and exemestane show improved efficacies against breast cancer and could reduce the side effects of tamoxifen [8,9,10,11]. However, they may also cause some other side effects, such as osteoporosis and alterations in lipid profiles [12,13]. 

Natural products have long been considered as an important source of chemopreventive and chemotherapeutic agents. The large number of bioactive natural products will offer unprecedented opportunities for finding novel small molecules possessing both efficacy and safety targeting the aromatase. More and more natural products such as flavonoids [14], coumarins [15], sesquiterpenes [16] and polyphenols [17] have been proved to possess potent aromatase inhibiting activities. *Corydalis yanhusuo* is a well-known traditional Chinese medicine (TCM) and has been used to promote blood circulation, reinforce vital energy and alleviate various kinds of pain for a long history [18,19]. The mainly bioactive chemical constituents in *C. yanhusuo* are tertiary and quaternary isoquinoline alkaloids [20,21,22]. Considering the previous studies indicating that *C. yanhusuo* could effectively inhibit human breast cancer cells [23] and alkaloids possessed aromatase inhibiting activities [24], in this study, we established an ultrafiltration liquid chromatography tandem mass spectrometry (LC-MS) method to screen the potential aromatase binding agents from the dichloromethane extract of *C. yanhusuo*.

## 2. Results and Discussion

### 2.1. HPLC-DAD-MS/MS analysis of the dichloromethane extract of C. yanhusuo

**Figure 1:**
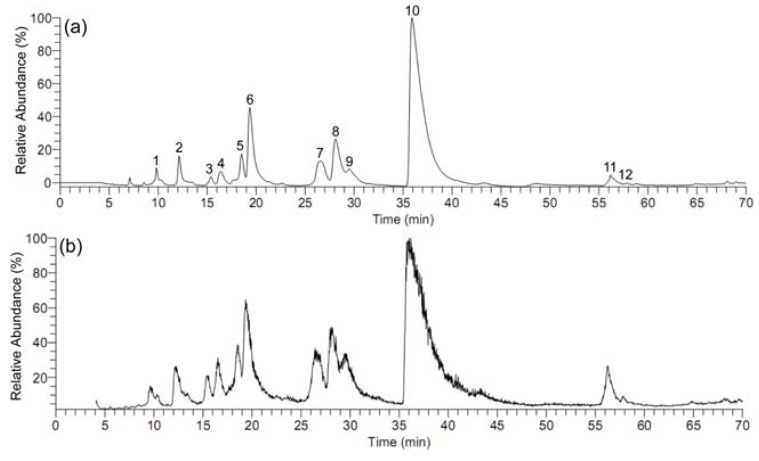
The HPLC-DAD chromatogram (a) and the total ion current (TIC) chromatogram (b) of the dichloromethane extract of *C. yanhusuo*.

The HPLC-DAD (Diode Array Detector) chromatogram and the total ion current (TIC) chromatogram of the dichloromethane extract of *C. yanhusuo *are shown in Figure 1. 

**Figure 2:**
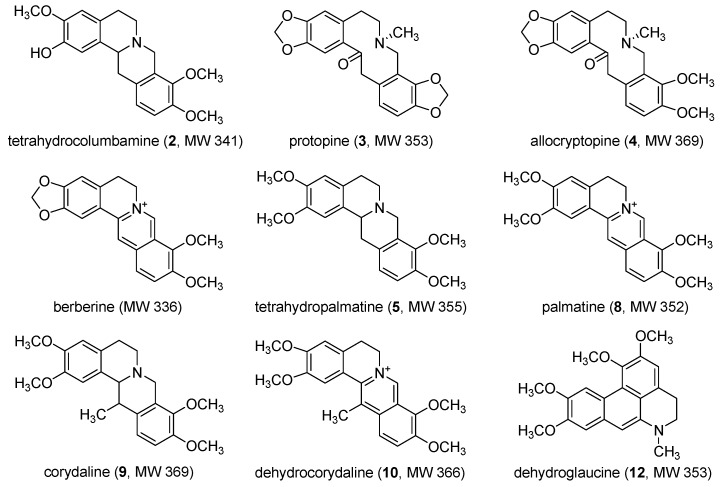
Chemical structures of isolated compounds from the dichloromethane extract of *C. yanhusuo*.

The positive ion mode of electrospray ionization (ESI) was selected for all subsequent MS analysis of the constituents in the dichloromethane extract of *C. yanhusuo* because the protonated molecules of these compounds in positive ion mode were well responsed. As shown in Figure 1, under the optimized HPLC-DAD-MS/MS condition, twelve constituents in *C. yanhusuo* were well separated. Then we isolated nine reference compounds from the dichloromethane extract of *C. yanhusuo* (Figure 2). 

Their structures were elucidated by NMR spectroscopic analysis and comparison with the literature data [25,26,27,28]. The ^1^H- and ^13^C-NMR spectra of each reference compound was shown in supplementary material. By comparing the MS^2^ fragments and the ultraviolet (UV) absorptions of the peaks in the dichloromethane extract of *C. yanhusuo* with those of the isolated reference compounds, we identified eight peaks in the HPLC-DAD chromatogram in Figure a. One of the isolated reference compounds, berberine, was not observed in the HPLC-DAD chromatogram and the TIC chromatogram of the dichloromethane extract of *C. yanhusuo* because of its low content. The data of retention time, MS^2^ fragment and the special UV wavelength of the constituents detected in the dichloromethane extract of *C. yanhusuo *were listed in Table 1.

**Table 1:** Peak assignments for analysis of the dichloromethane extract of *C. yanhusuo*.

No.	t_R_	Identification	[M+H]^+^ *m/z*	MS^2^ *m/z*	UV λ_max_ (nm)
1	9.72	unknown	356	326,192	209, 229, 276
2	12.11	tetrahydrocolumbamine	342	178	208, 229, 282
3	15.45	protopine	354	320,206,188	209, 237, 289
4	16.52	allocryptopine	370	352,324,320,188	208, 225, 284
5	18.59	tetrahydropalmatine	356	320,192	209, 229, 282
6	19.29	unknown	356	325,279	225, 281, 302
7	26.61	unknown	352	337,279	229, 266, 336
8	28.09	palmatine	352	336,294,278	228, 273, 344
9	29.57	corydaline	370	352,336,294,192	208, 229, 282
10	36.02	dehydrocorydaline	366	336,292	229, 272, 335
11	56.09	unknown	366	336,308	215
12	57.83	dehydroglaucine	354	336,292	233, 258, 320

We noticed that many constituents in the dichloromethane extract of *C. yanhusuo* had the same molecular weights, but different retention times. Peaks 1, 5 and 6 obtained the same ions at *m/z* 356 and their retention times were 9.72, 18.59 and 19.29 min respectively. We identified peak 5 as tetrahydropalmatine, according to the same MS^2^ fragment and UV spectrum with the isolated reference compound. However, when came to peak 1 and 6, since no reference compounds were available for comparison, it was difficult to characterize their accurate structures using only the MS^2^ fragments and the UV spectra. The same situation occurred in the identification of peaks 7 and 8, which had the same ions at *m/z* 352. Peaks 8 and 12 were identified as palmatine and dehydroglaucine by comparing the MS^2^ fragments and the UV spectra with those of the isolated reference compounds.

### 2.2. Ultrafiltration LC-MS screening for aromatase binding agents from the dichloromethane extract of C. yanhusuo

**Figure 3:**
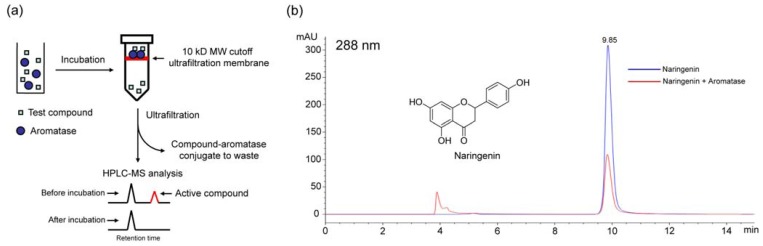
Ultrafiltration LC-MS screening for the aromatase binding activity of naringenin. (A) Schematic of the ultrafiltration LC-MS screening method. (B) HPLC chromatograms before (blue) and after (red) incubation of naringenin with aromatase.

In previous studies, ultrafiltration combined with LC-MS could be used to investigate the ligands to some macromolecular targets, such as adenosine deaminase [29,30], cyclooxygenase-2 [31] and estrogen receptors [32,33]. Herein, we used this screening method to discover aromatase binding agents from the dichloromethane extract of *C. yanhusuo*. The schematic of the ultrafiltration LC-MS screening method is shown in Figure 3a. First, compounds in the extract were allowed to bind to aromatase, and then ultrafiltration was used to separate the aromatase-ligand conjugates from the compounds that did not bind to aromatase. Finally, LC-MS was employed to analyze the ultrafiltrate in which the peak area of aromatase binding agent would decrease or disappear compared with that of the control, and thus specific aromatase binding agents could be identified clearly. To validate the assay, we first chose naringenin as the positive control, which had been proved to possess aromatase inhibiting activity in previous studies [34,35], the result is shown in Figure 3b.

**Figure 4:**
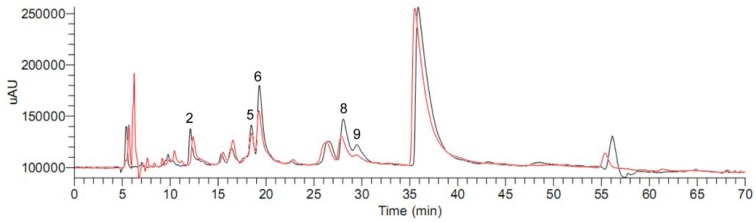
HPLC chromatograms before (black) and after (red) incubation of the dichloromethane extract of *C. yanhusuo* with aromatase.

Subsequently, the dichloromethane extract of *C. yanhusuo* was analyzed by the ultrafiltration LC-MS screening method to discover the potential aromatase binding agents. As shown in Figure 4, after incubation of the dichloromethane extract of *C. yanhusuo* with aromatase, the area of peaks 2, 5, 6, 8 and 9 decreased by 21.0%, 22.5%, 41.0%, 45.8% and 74.2% respectively. 

**Figure 5:**
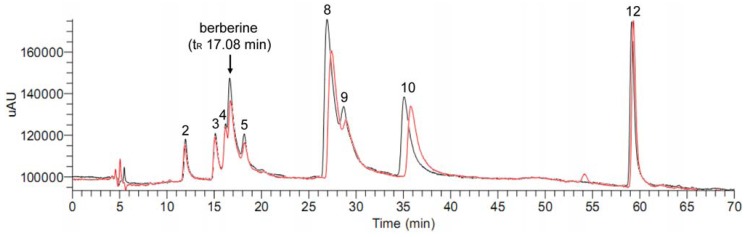
HPLC chromatograms before (black) and after (red) incubation of the mixed reference compounds with aromatase.

The data of the peak areas that compounds before and after incubating with aromatase is listed in Table 2. Except for peak 6 that could not be identified due to the lack of reference compound, the other peaks 2, 5, 8 and 9 represented tetrahydrocolumbamine, tetrahydropalmatine, palmatine and corydaline respectively. Then, nine isolated reference alkaloids were mixed and their aromatase binding activities tested using the same screening method as described above to validate our results. As shown in Figure 5, after incubation of the mixed reference compounds with aromatase, the area of peaks 2, 5, 8, 9 and 10 decreased by 7.0%, 18.2%, 23.6%, 36.5% and 18.5% respectively (Table 2). In addition, the area of the peak at 17.08 min, corresponding to the isolated alkaloid berberine which could not be observed in Figure 1 and Figure 4, also decreased by 27.8%. The results of the mixed sample were coincident with those of the extract except for peak 10, which was unchanged in the extract but decreased in the mixed sample. This could be explained that the relative content of dehydrocorydaline (peak 10) in the dichloromethane extract of *C. yanhusuo* was much higher than that in the mixed sample, since the binding site of aromatase was limited, the few binding dehydrocorydaline could not result in the significant difference between incubation with aromatase and the control group.

**Table 2:** Peak areas of compounds before and after incubating with aromatse.

Peak no.	Dichloromethane extract			Mixed reference compounds		
	Before (mAU·min)	After (mAU·min)	Decrease (%)	Before (mAU·min)	After (mAU·min)	Decrease (%)
**2**	770.103	608.381	21.0	464.950	432.403	7.0
**5**	715.843	554.778	22.5	297.321	243.208	18.2
**6**	2257.137	1331.711	41.0	-	-	-
**8**	1558.298	844.598	45.8	4329.332	3307.610	23.6
**9**	338.888	87.433	74.2	1306.109	829.379	36.5
**10**	-	-	-	2202.736	1795.230	18.5
berberine	-	-	-	857.387	619.033	27.8

**Figure 6:**
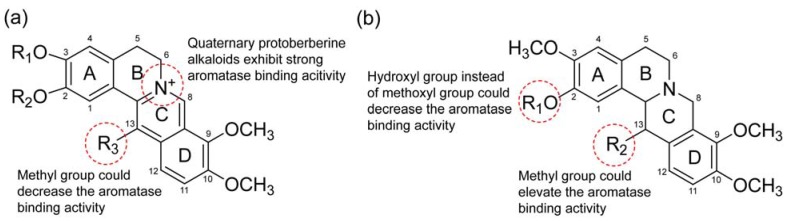
The possible structure-binding activity relationships of the alkaloids from the dichloromethane extract of *C. yanhusuo*.

Comparing the chemical structures of the six active alkaloids above, all the quaternary protoberberine alkaloids (berberine, palmatine and dehydrocorydaline) show strong aromatase binding activities (Figure 6a), indicating the importance of quaternary ammonium group in the interaction between the alkaloids and aromatase. When C-13 position of C ring is substituted by methyl group, the aromatase binding activity was slightly decrease (the decrease of peak area of palmatine is 5.1% higher than that of dehydrocorydaline, as shown in Figure 5). The potent binding might be the result of the participation of quaternary ammonium group in the hydrogen bonds interactions with the carboxylate oxygen atoms in protein residues. When comes to the tertiary protoberberine alkaloids, they also have aromatase binding activities. Comparing the structures of tetrahydrocolumbamine (peak 2), tetrahydropalmatine (peak 5) and corydaline (peak 9), it is demonstrated that the methoxyl group at C-2 position of A ring superseded by the hydroxyl group will decrease the aromatase binding activity, and the methyl group substituted at C-13 position of C ring will obviously increase the activity (Figure 6b). The other types of alkaloids such as the protopine analogs (protopine and allocryptopine) and the glaucine analogs (dehydroglaucine) possessed no aromatase binding activities in this study.

## 3. Experimental

### 3.1. Apparatus

Prep. HPLC: Agilent-1100 system; photodiode array detector; Zorbax-C_18_ column (250 × 21.2 mm, 7 μm, Agilent Technologies, USA). ^1^H- and ^13^C-NMR: Bruker Ultrashield Plus 500 MHz spectrometer. HPLC: Agilent 1100 HPLC system (Waldbronn, Germany) equipped with a Zorbax SB-C_18_ column (250 × 4.6 mm, 5 μm, Agilent Technologies, USA). ESI-MS: Thermo Finnigan LCQ Deca XP^plus^ ESI ion trap mass spectrometer (San Jose, CA, USA) in positive ion mode. The MS operating parameters were as follows: collision gas, ultra-high-purity helium (He); nebulizing gas, high-purity nitrogen (N_2_); ion spray voltage, -4.5 kV; sheath gas (N_2_), 5 arbitrary units; capillary temperature, 275 °C; capillary voltage, -15 V; tube lens offset voltage, -30 V. The collision energy for collision-induced dissociation (CID) was between 30% and 45%, and the isolation width of precursor ions was 3.0 Th.

### 3.2. Material and reagents

The air-dried tubers of *C. yanhusuo* were collected in Pan’an, Zhejiang province, People’s Republic of China, in March 2008. The plant material was identified by the authors and a voucher specimen (No. 080401) had been deposited in the herbarium of the School of Pharmaceutical Sciences, Zhejiang University. Aromatase was purchased from BD Biosciences (San Jose, CA, USA). HPLC-grade methanol (Merck, Darmstadt, Germany) and formic acid (Tedia, Fairfield, OH, USA) were utilized for the HPLC analysis. Deionized water was purified using a Milli-Q system (Millipore, Bedford, MA, USA). All the other chemicals and solvents were of analytical-reagent grade. Nine reference compounds including tetrahydrocolumbamine, protopine, allocryptopine, berberine, tetrahydropalmatine, palmatine, corydaline, dehydrocorydaline and dehydroglaucine were isolated from the dichloromethane fraction of the 95% ethanol extract of *C. yanhusuo*. Their structures were elucidated by NMR spectroscopic analysis and comparison with the literature data. Purities of all the reference compounds were greater than 95% according to HPLC analysis.

### 3.3. HPLC conditions

HPLC analysis was performed on an Agilent 1100 HPLC instrument coupled to a binary pump, a diode array detector (DAD), an autosampler and a column thermostat. The sample was analyzed on a Zorbax SB-C_18_ column (250 × 4.6 mm, 5 μm, Agilent Technologies, USA). A linear gradient elution of A (10 mM ammonium acetate solution, pH was adjusted to 3.5 by formic acid) and B (methanol) was used according to the following profile: 0–40 min, 38% B; 40–55 min, 38-50% B; 55–65 min, 50–70% B and maintained 70% B during the next 10 min. The flow rate was 0.5 mL/min and the column temperature was set at 30 °C. The injection volume was 10 μL. The UV spectra were recorded from 190 to 400 nm.

### 3.4. Ultrafiltration LC-MS screening

Nine isolated reference alkaloids were mixed and dissolved in methanol as the reference compound stock solution (1 mM for each compound). The dichloromethane fraction of the 95% ethanol extract of *C. yanhusuo* was dissolved in methanol to obtain the extract stock solution (20 mg/mL). The extract stock solution and the reference compounds stock solution were incubated with 50 nM aromatase for 30 min at 37 °C in a total volume of 200 µL of deionized water (The final concentration of the extract stock solution and each reference compound were 2 mg/mL and 100 μM, respectively). After incubation, the solution was filtered though a Microcon (Millipore, Bedford, MA) YM-10 centrifugal filter containing a regenerated cellulose ultrafiltration membrane with a 10,000 MW cutoff by centrifugation at 12,000 g for 30 min at room temperature. The ultrafiltrate was transferred into HPLC vials and analyzed by LC-MS. For comparison, the two above mentioned sample solutions were prepared in an identical manner except for the use of aromatase. Naringenin was chosen as the positive control (The final concentration of naringenin in incubation solution was 100 μM).

## 4. Conclusions

In this paper, we identified nine alkaloids from the dichloromethane extract of *C*.* yanhusuo* and tested their aromatase binding activities using an ultrafiltration LC-MS screening method. The results showed that the quaternary protoberberine alkaloids and the tertiary protoberberine alkaloids had aromatase binding activities, while the protopine analogs and the glaucine analogs had no such activities. Based on the results of the study, the quaternary protoberberine alkaloids and the tertiary protoberberine alkaloids certainly merit continued and comparative pharmacological study for the future.

## Supplementary Materials

Supplementary materials can be seen at http://www.mdpi.com/1420-3049/15/5/3556/S1.

## References

[1] Bulun S.E., Lin Z., Imir G., Amin S., Demura M., Yilmaz B., Martin R., Utsunomiya H., Thung S., Gurates B., Tamura M., Langoi D., Deb S. (2005). Regulation of aromatase expression in estrogen-responsive breast and uterine disease: from bench to treatment. Pharmacol. Rev..

[2] Santen R.J. (2003). Inhibition of aromatase: insights from recent studies. Steroids.

[3] Fisher B., Costantino J.P., Wickerham D.L., Redmond C.K., Kavanah M., Cronin W.M., Vogel V., Robidoux A., Dimitrov N., Atkins J., Daly M., Wieand S., Tan-Chiu E., Ford L., Wolmark N. (1998). Tamoxifen for prevention of breast caner: Report of the National Surgical Adjuvant Breast and Bowel Project P-1 Study. J. Natl. Cancer Inst..

[4] Brueggemeier R.W., Hackett J.C., Diaz-Cruz E.S. (2005). Aromatase inhibitors in the treatment of breast cancer. Endocr. Rev..

[5] Evans C.T., Ledesma D.B., Schulz T.Z., Simpson E.R., Mendelson C.R. (1986). Isolation and characterization of a complementary DNA specific for human aromatase-system cytochrome P450 mRNA. Proc. Natl. Acad. Sci. USA.

[6] Brueggemeier R.W. (2006). Update on the use of aromatase inhibitors in breast cancer. Expert Opin. Pharmacother..

[7] Kendall A., Dowsett M. (2006). Novel concepts for the chemoprevention of breast cancer through aromatase inhibition. Endocr. Relat. Cancer.

[8] Wong Z.W., Ellis M.J. (2004). First-line endocrine treatment of breast cancer: aromatase inhibitor or antioestrogen?. Brit. J. Cancer.

[9] Milla-Santos A., Milla L., Portella J., Rallo L., Pons M., Rodes E., Casanovas J., Puig-Gali M. (2003). Anastrozole versus tamoxifen as first-line therapy in postmenopausal patients with hormone-dependent advanced breast cancer: a prospective, randomized, phase III study. Am. J. Clin. Oncol..

[10] Arora A., Potter J.F. (2004). Aromatase inhibitors: current indications and future prospects for treatment of postmenopausal breast cancer. J. Am. Geriatr. Soc..

[11] Goss P.E. (1999). Risks versus benefits in the clinical application of aromatase inhibitors. Endocr. Relat. Cancer.

[12] Gnant M. (2006). Management of bone loss induced by aromatase inhibitors. Cancer Invest..

[13] Chlebowski R.T., Anderson G.L., Geller M., Col N. (2006). Coronary heart disease and stroke with aromatase inhibitor, tamoxifen, and menopausal hormone therapy use. Clin. Breast Cancer.

[14] Lee D., Bhat K.P., Fong H.H., Farnsworth N.R., Pezzuto J.M., Kinghorn A.D. (2001). Aromatase inhibitors from *Broussonetia papyrifera*. J. Nat. Prod..

[15] Chen S., Cho M., Karlsberg K., Zhou D., Yuan Y.C. (2004). Biochemical and biological characterization of a novel anti-aromatase coumarin derivative. J. Biol. Chem..

[16] Blanco J.G., Gil R.R., Alvarea C.I., Patrito L.C., Genti-Raimondi S., Flury A. (1997). A novel activity for a group of sesquiterpene lactones: inhibition of aromatase. FEBS Lett..

[17] Wang Y., Lee K.W., Chan F.L., Chen S., Leung L.K. (2006). The red wine polyphenol reseratrol displays bilevel inhibition on aromatase in breast cancer cells. Toxicol. Sci..

[18] Kadohama N., Shintani K., Osawa Y. (1993). Tobacco alkaloid derivatives as inhibitors of breast cancer aromatase. Cancer Lett..

[19] Ng P.C., Ho D.D., Ng K.H., Kong Y.C., Cheng K.F., Stone G. (1994). Mixed estrogenic and anti-estrogenic activities of yuehchukene-a bis-indole alkaloid. Eur. J. Pharmacol..

[20] Ding B., Zhou T., Fan G., Hong Z., Wu Y. (2007). Qualitative and quantitative determination of ten alkaloids in traditional Chinese medicine *Corydalis yanhusuo* W.T. Wang by LC-MS/MS and LC-DAD. J. Pharm. Biomed. Anal..

[21] Ma Z.Z., Xu W., Jensen N.H., Roth B.L., Liu-Chen L.Y., Lee D.Y. (2008). Isoquinoline alkaloids isolated from *Corydalis yanhusuo* and their binding affinities at the dopamine D1 receptor. Molecules.

[22] Ou J., Kong L., Pan C., Su X., Lei X., Zou H. (2006). Determination of DL-tetrahydropalmatine in *Corydalis yanhusuo* by L-tetrahydropalmatine imprinted monolithic column coupling with reversed-phase high performance liquid chromatography. J. Chromatogr. A.

[23] Gao J.L., Shi J.M., He K., Zhang Q.W., Li S.P., Lee S.M., Wang Y.T. (2008). Yanhusuo extract inhibits metastasis of breast cancer cells by modulating mitogen-activated protein kinase signaling pathways. Oncol. Rep..

[24] Nobuyuki K., Keiji S., Yoshio O. (1993). Tobacco alkaloid derivatives as inhibitors of breast cancer aromatase. Cancer Lett..

[25] Grycova L., Dostal J., Marek R. (2007). Quaternary protoberberine alkaloids. Phytochemistry.

[26] Cutter P.S., Miller R.B., Schore N.E. (2002). Synthesis of protoberberines using a silyl–directed Pictet-Spengler cyclization. Tetrahedron.

[27] Cushman M., Dekow F.W. (1978). A total synthesis of corydaline. Tetrahedron.

[28] Wada Y., Kaga H., Uchiito S., Kumazawa E., Tomiki M., Onozaki Y., Kurono N., Tokuda M., Ohkuma T., Orito K. (2007). On the synthesis of protopine alkaloids. J. Org. Chem..

[29] Van Breemen R.B., Huang C.R., Nikolic D., Woodbury C.P., Zhao Y.Z., Venton D.L. (1997). Pulsed ultrafiltration mass spectrometry: A new method for screening combinatorial libraries. Anal. Chem..

[30] Zhao Y.Z., van Breemen R.B., Nikolic D., Huang C.R., Woodbury C.P., Schilling A., Venton D.L. (1997). Screening solution-phase combinatorial libraries using pulsed ultrafiltration/electrospray mass spectrometry. J. Med. Chem..

[31] Nikolic D., Habibi-Goudarzi S., Corley D.G., Gafner S., Pezzuto J.M., van Breemen R.B. (2000). Evalution of cyclooxygenase-2 inhibitors using pulsed ultrafiltration mass spectrometry. Anal. Chem..

[32] Liu J., Burdette J.E., Xu H., Gu C., van Breemen R.B., Bhat K.P.L., Booth N., Constantinou A.I., Pezzuto J.M., Fong H.H.S., Farnsworth N.R., Bolton J.L. (2001). Evaluation of estrogenic activity of plant extracts for the potential treatment of menopausal symptoms. J. Agric. Food Chem..

[33] Sun Y., Gu C., Liu X., Liang W., Yao P., Bolton J.L., van Breemen R.B. (2005). Ultrafiltration tandem mass spectrometry of estrogens for characterization of structure and affinity for human estrogen receptors. J. Am. Soc. Mass Spectrom..

[34] Le Bail J.C., Laroche T., Marre-Fournier F., Habrioux G. (1998). Aromatase and 17-hydroxysteroid dehydrogenase inhibition by flavonoids. Cancer Lett..

[35] Sanderson J.T., Hordijk J., Denison M.S., Springsteel M.F., Nantz M.H., van den Berg M. (2004). Induction and inhibition of aromatase (CYP19) activity by natural and synthetic flavonoid compounds in H295R human adrenocortical carcinoma cells. Toxicol. Sci..

