# Supplementary material for: Characterization of Aromatase Binding Agents from the Dichloromethane Extract of Corydalis yanhusuo Using Ultrafiltration and Liquid Chromatography Tandem Mass Spectrometry
Source: Molecules. 2010 May 14;15(5):3556–66. doi: 10.3390/molecules15053556 (PMC6263280; doi:10.3390/molecules15053556)

Supplementary Material (^{1}H- and ^{13}C-NMR spectra of each reference compound)

Tetrahydrocolumbamine (**2**)

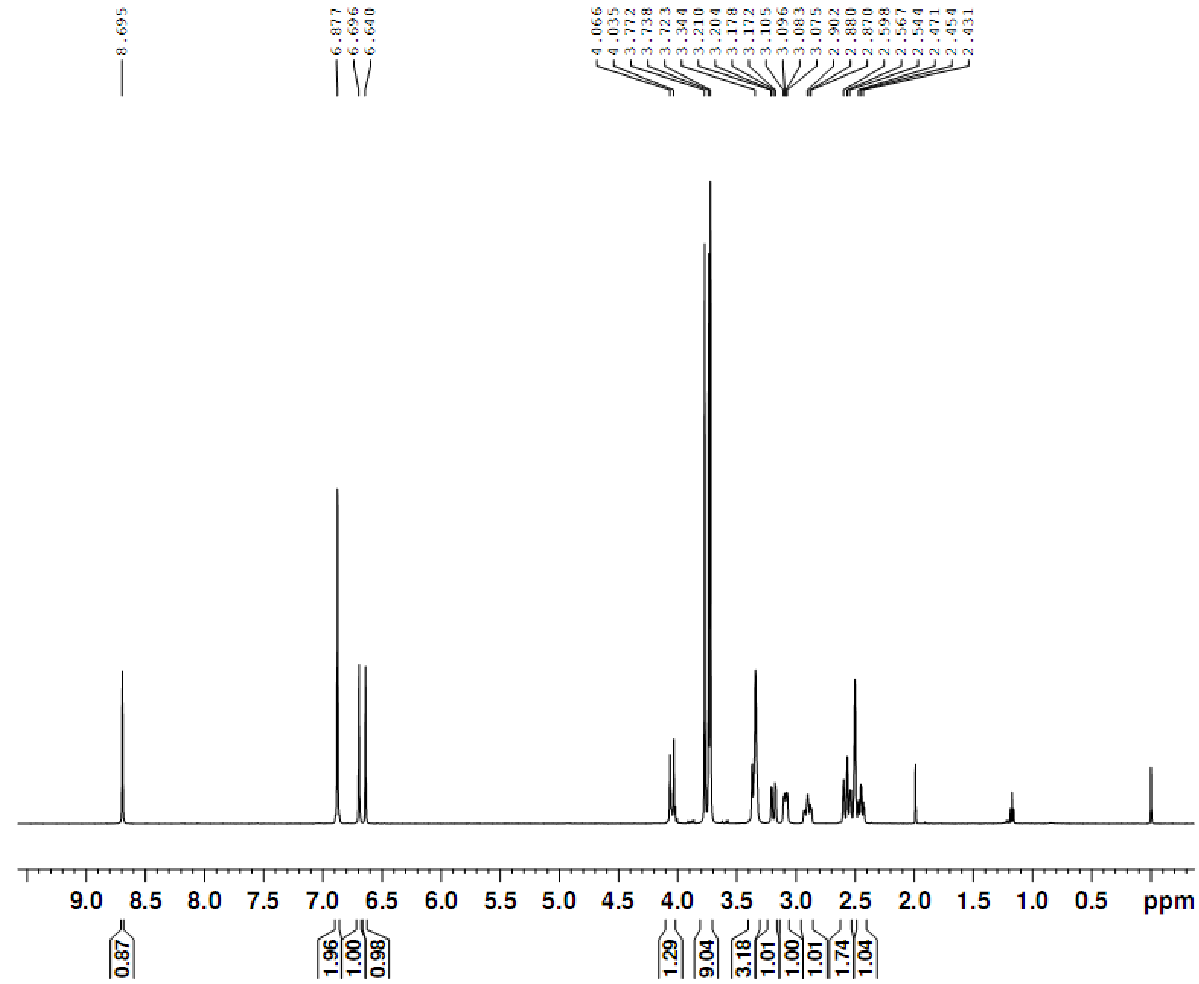

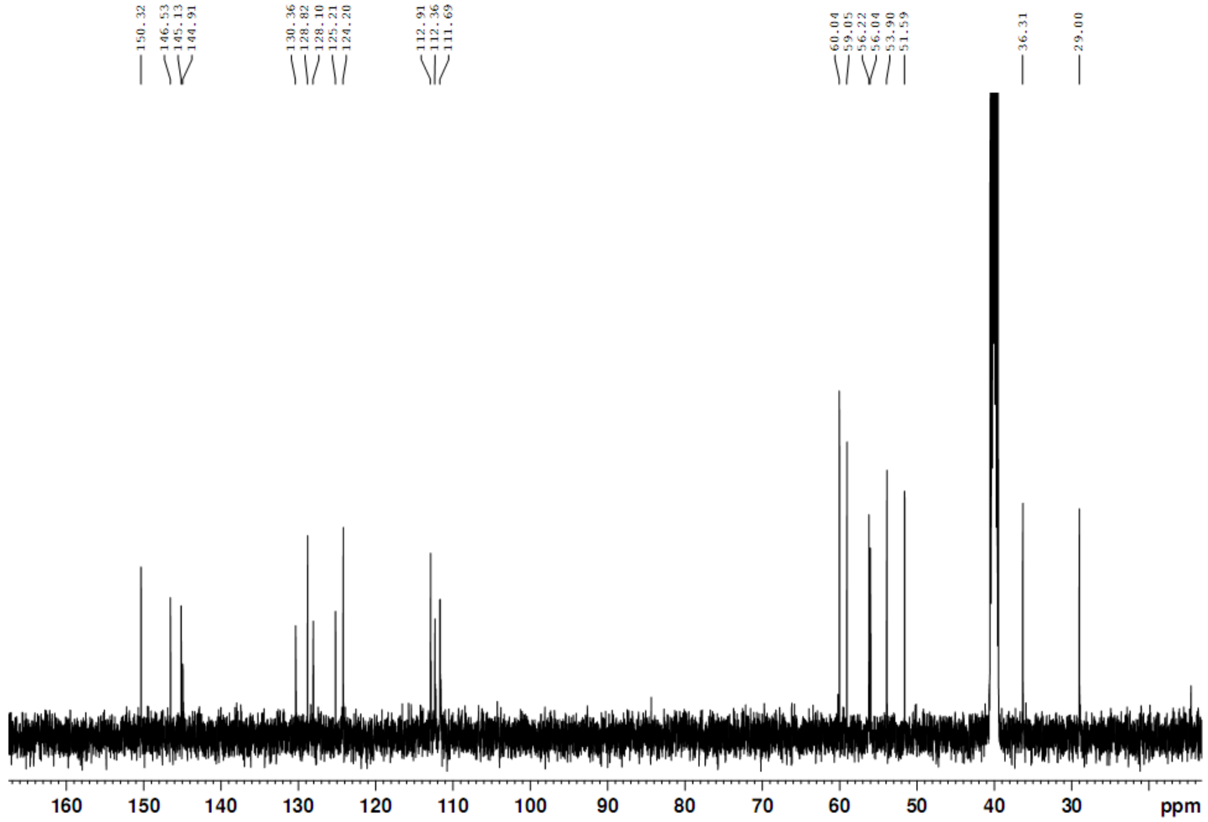

Protopine (**3**)

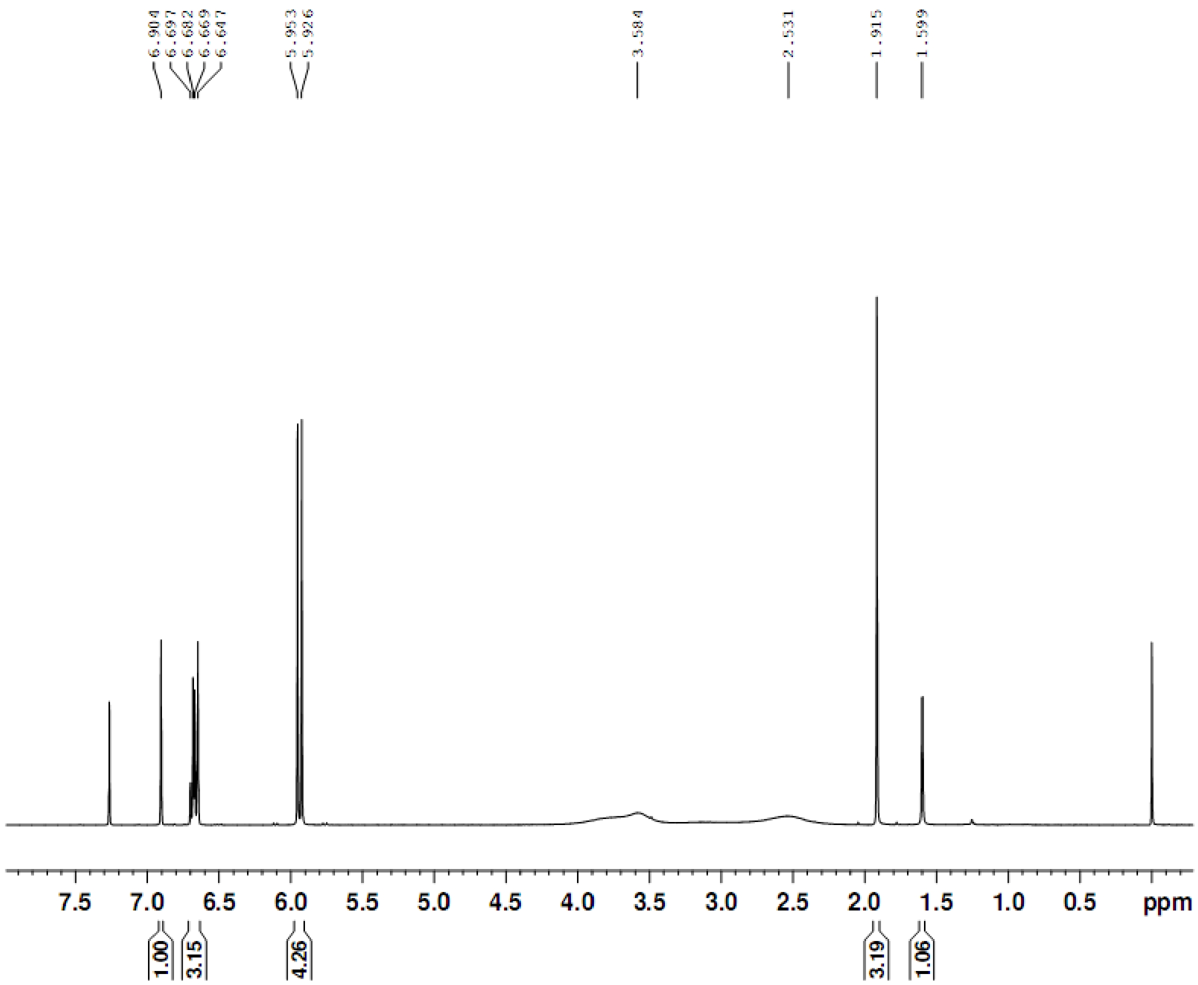
6.904
6.697
6.682
6.669
6.647
5.953
5.926
3.584
2.531
1.915
1.599
7.5 7.0 6.5 6.0 5.5 5.0 4.5 4.0 3.5 3.0 2.5 2.0 1.5 1.0 0.5 ppm
1.00
3.15
4.26
3.19
1.06

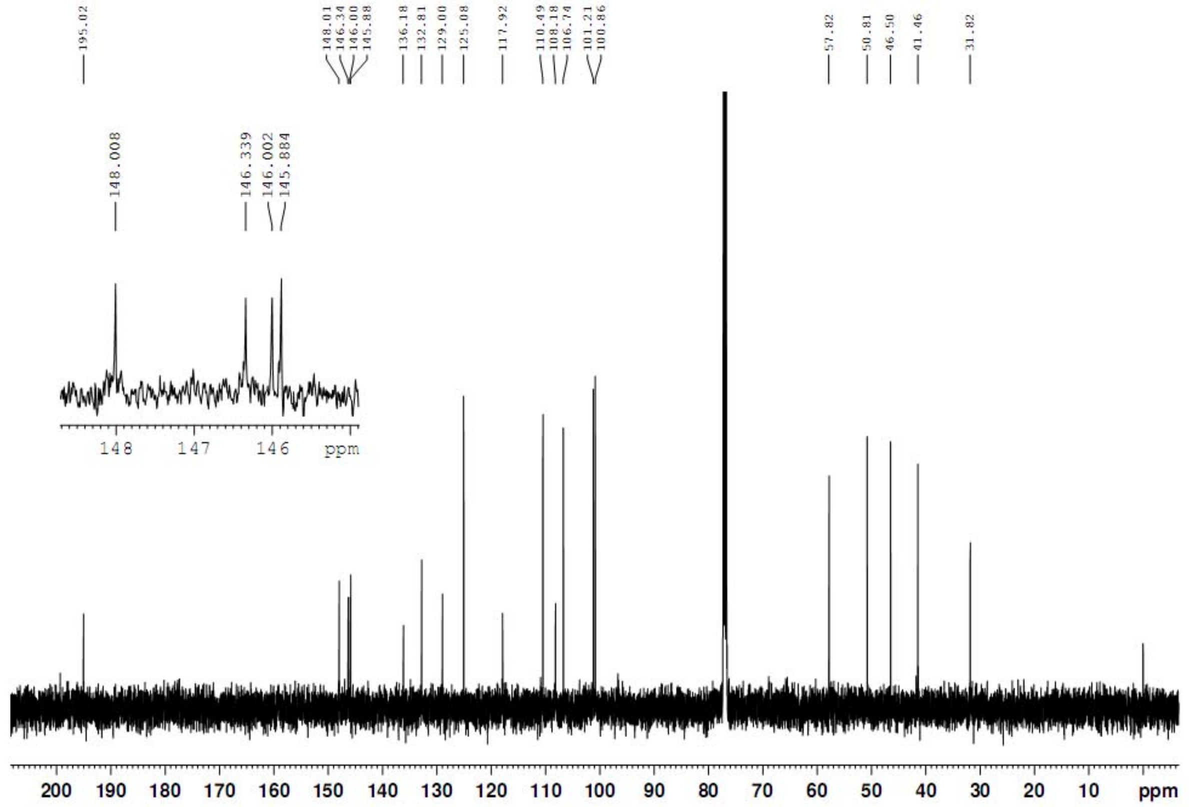
195.02
148.01
146.34
146.00
145.88
136.18
132.81
129.00
125.08
117.92
110.49
108.18
106.74
101.21
100.86
57.82
50.81
46.50
41.46
31.82
148.008
146.339
146.002
145.884
148 147 146 ppm
200 190 180 170 160 150 140 130 120 110 100 90 80 70 60 50 40 30 20 10 ppm

Allocryptopine (**4**)

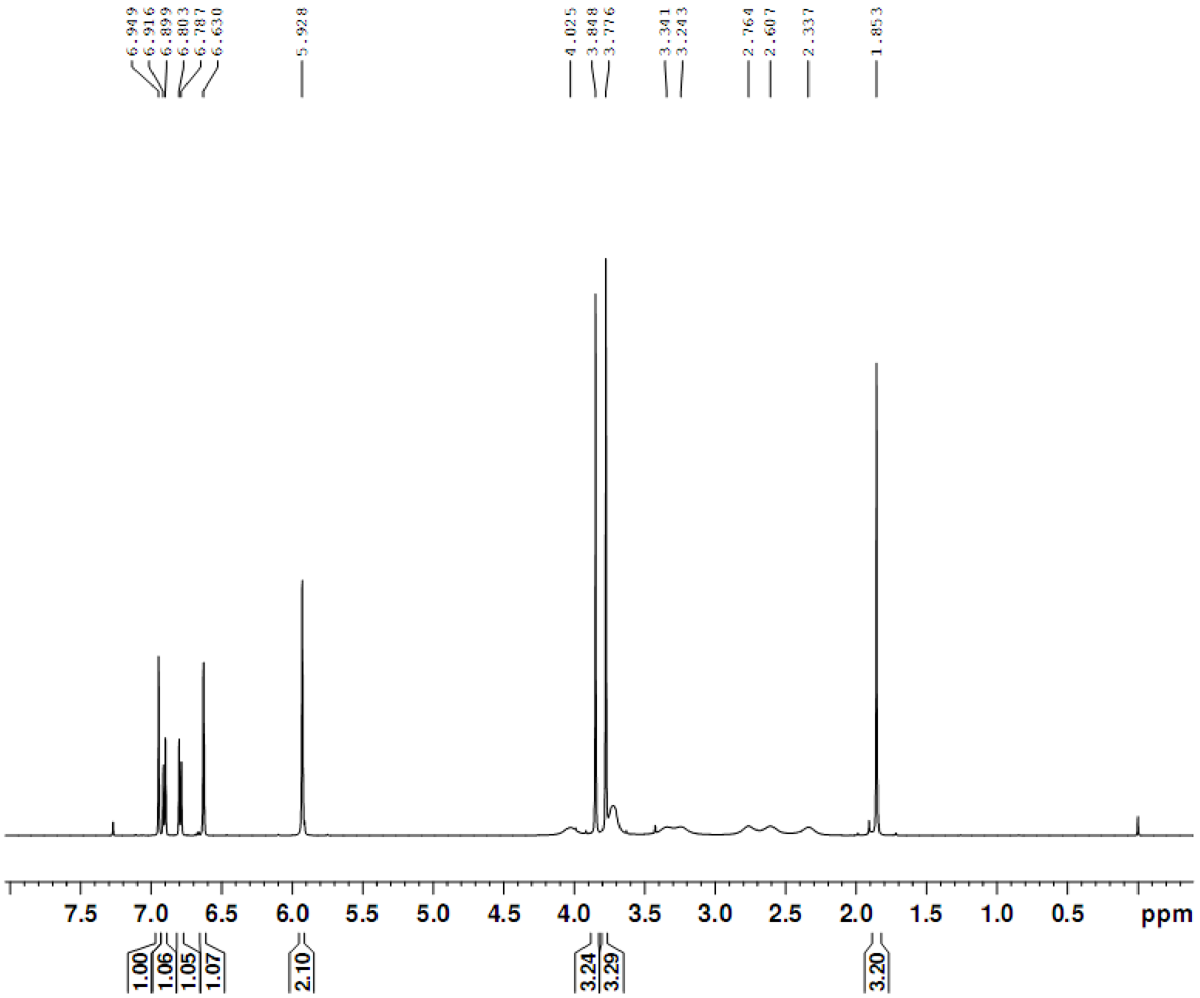

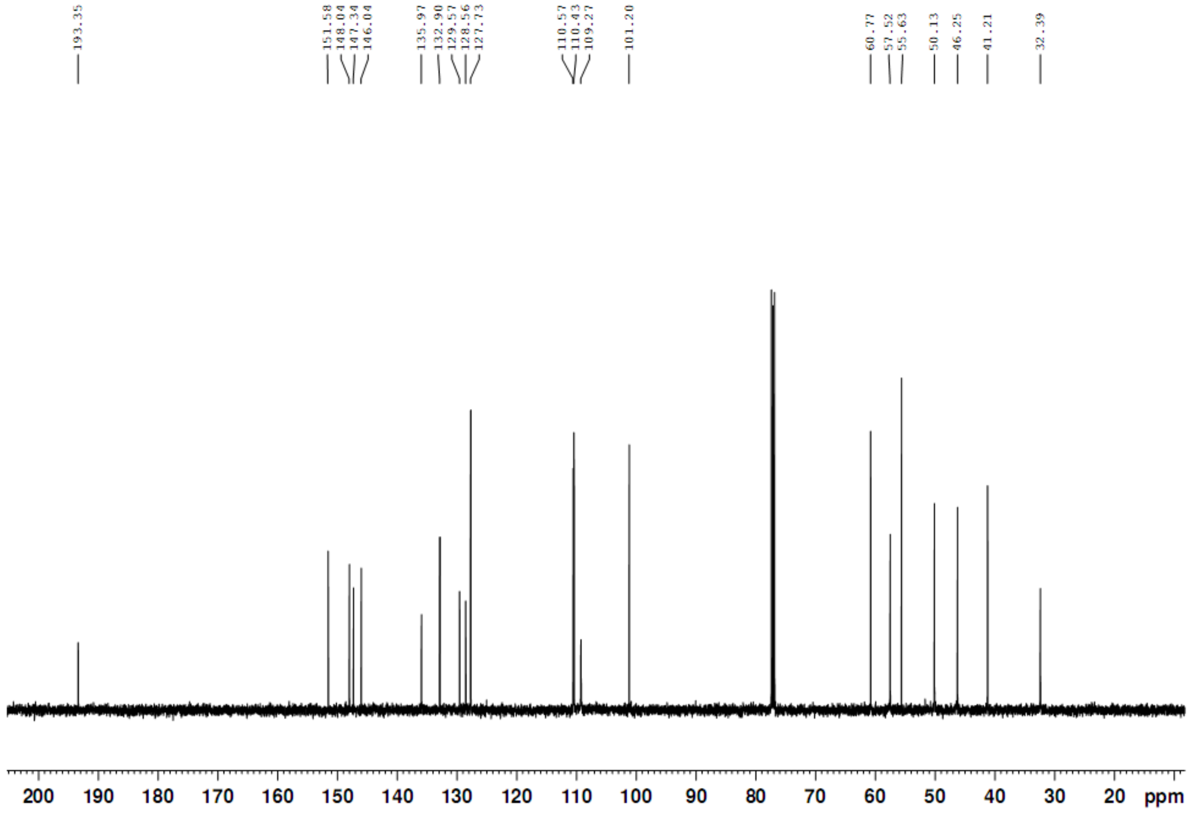

Berberine

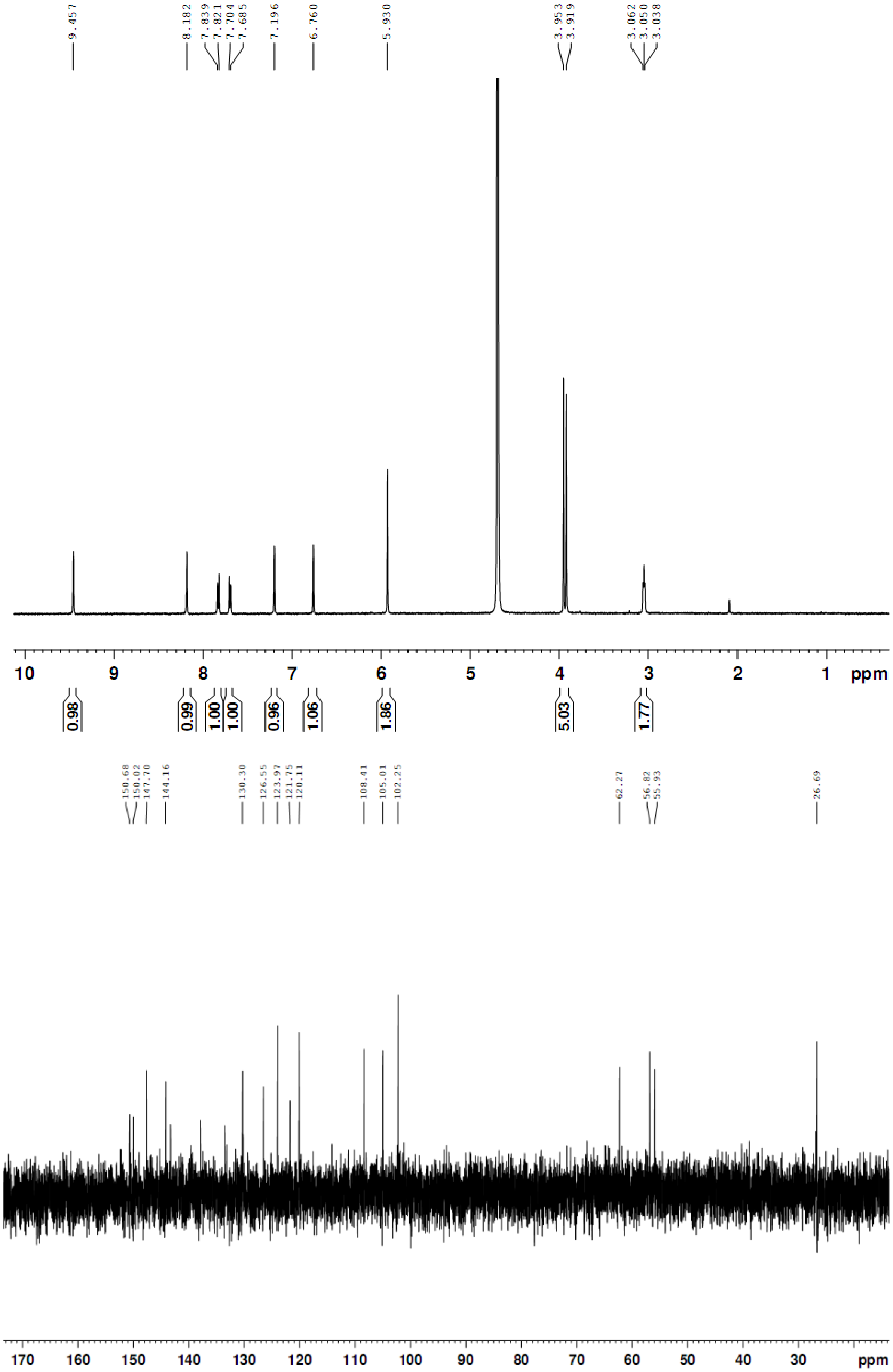
9.457
8.182
7.839
7.821
7.704
7.685
7.196
6.760
5.930
3.953
3.919
3.062
3.050
3.038
10
9
8
7
6
5
4
3
2
1
ppm
0.98
0.99
1.00
1.00
0.96
1.06
1.86
5.03
1.77
150.68
150.02
147.70
144.16
130.30
126.55
123.97
121.75
120.11
108.41
105.01
102.25
62.27
56.82
55.93
26.69
170
160
150
140
130
120
110
100
90
80
70
60
50
40
30
ppm

Tetrahydropalmatine (**5**)

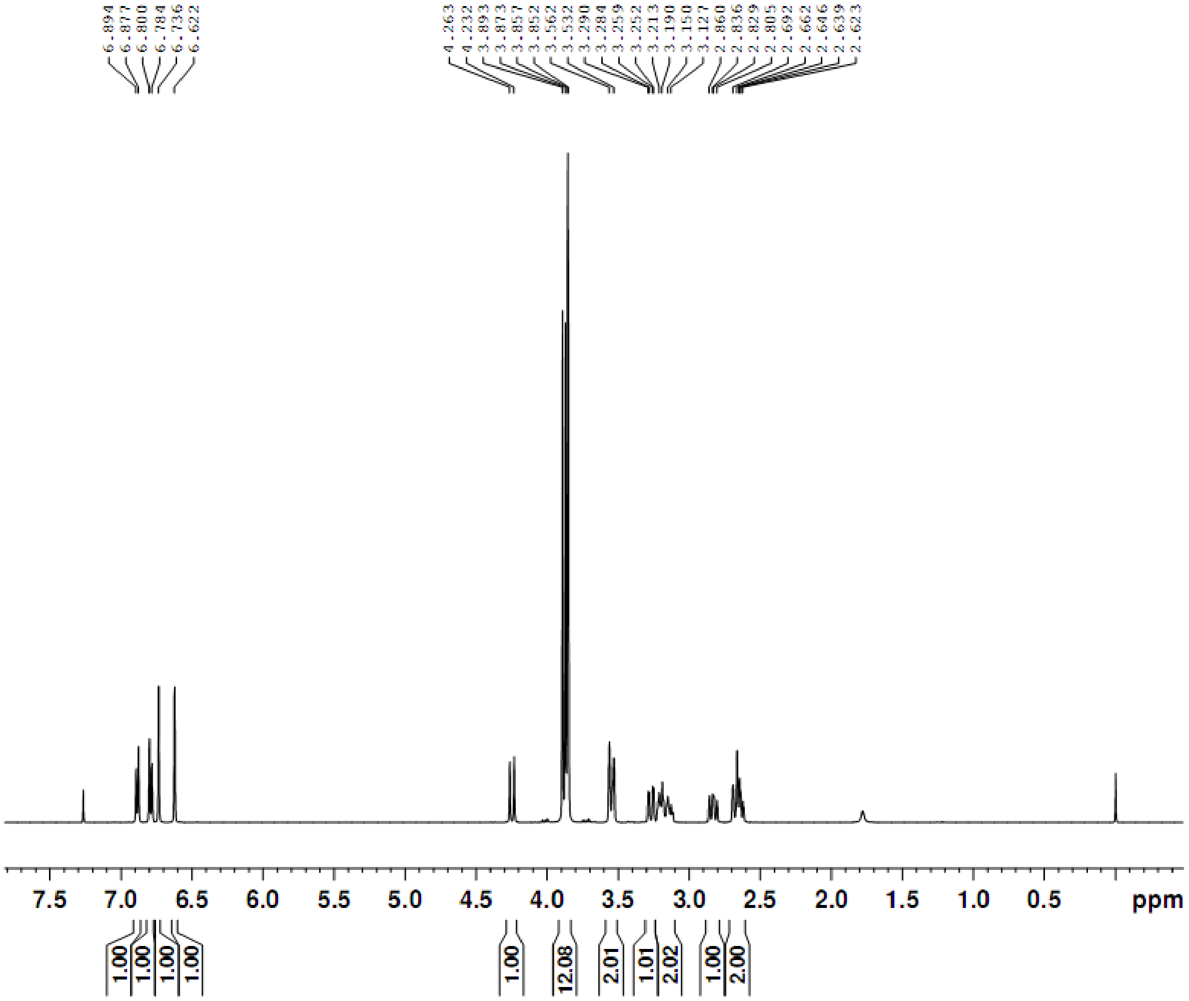

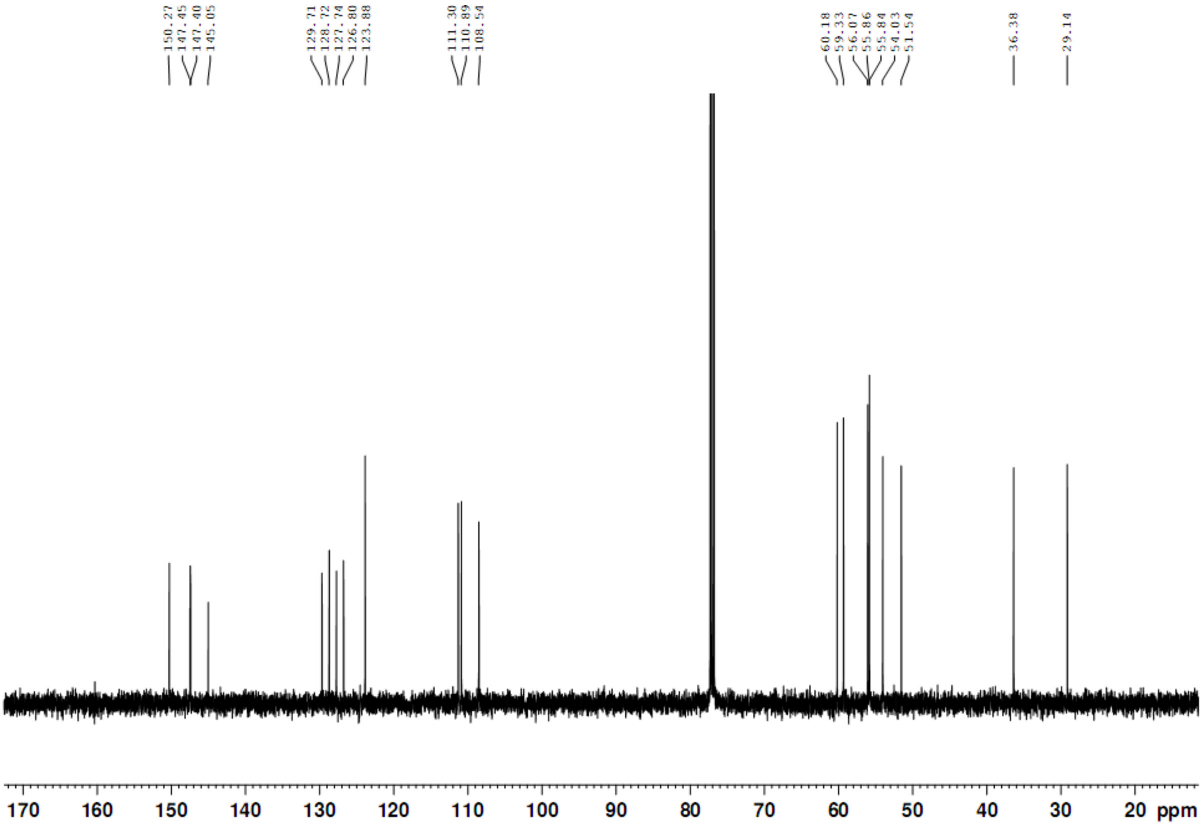

Palmatine (**8**)

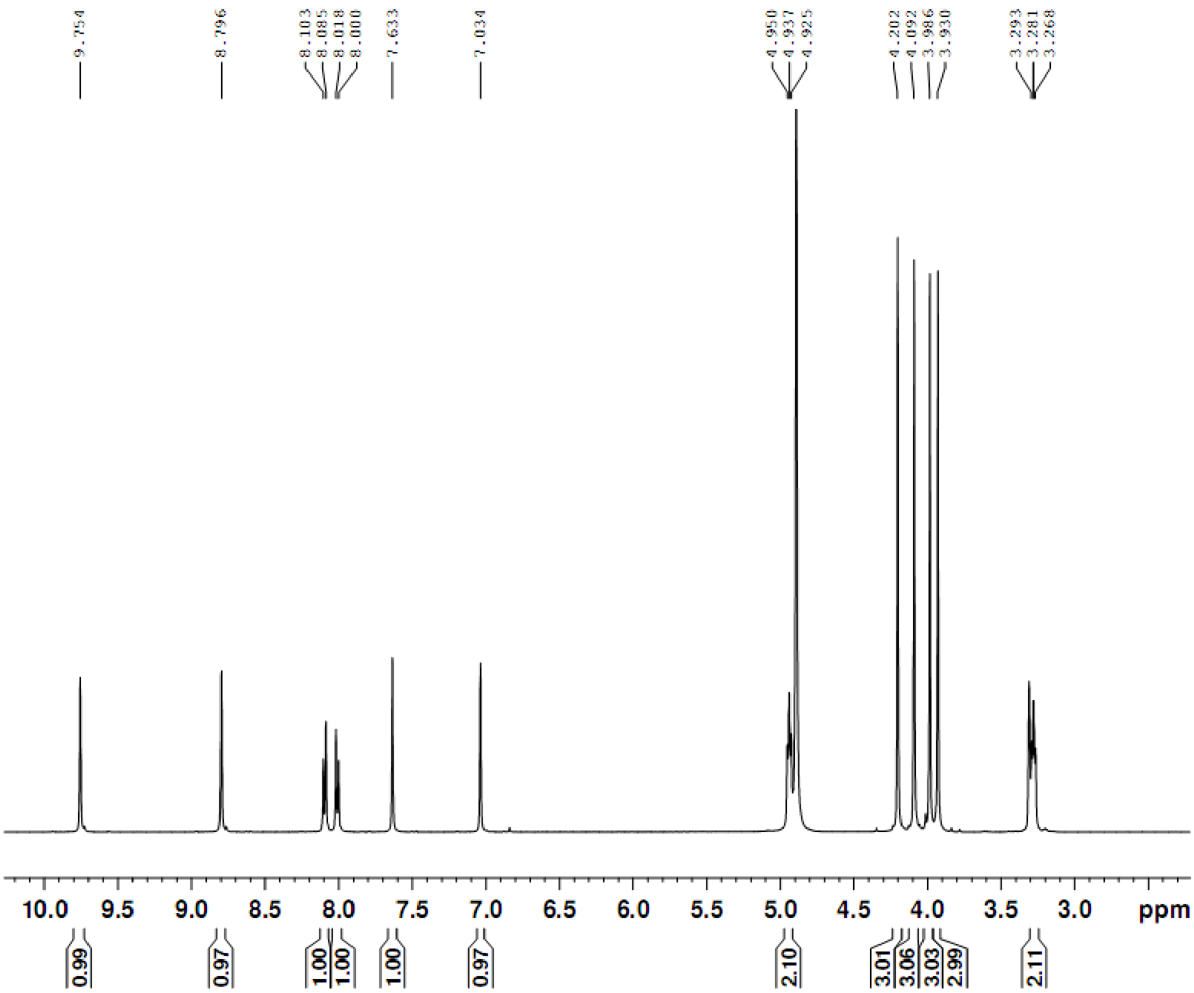
9.754
8.796
8.103
8.085
8.018
8.000
7.633
7.034
4.950
4.937
4.925
4.202
4.092
3.986
3.930
3.293
3.281
3.268
10.0 9.5 9.0 8.5 8.0 7.5 7.0 6.5 6.0 5.5 5.0 4.5 4.0 3.5 3.0 ppm
0.99
0.97
1.00
1.00
1.00
0.97
2.10
3.01
3.06
3.03
2.99
2.11

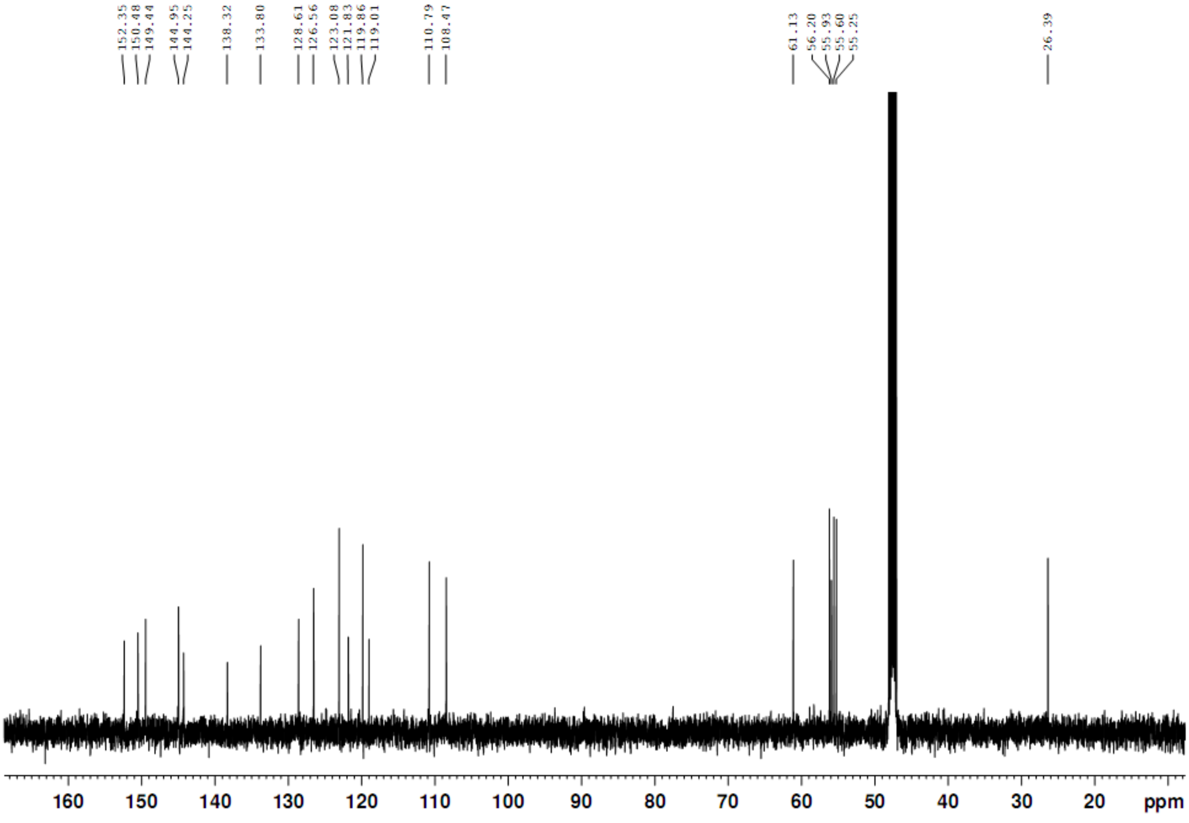
152.35
150.48
149.44
144.95
144.25
138.32
133.80
128.61
126.56
123.08
121.83
119.86
119.01
110.79
108.47
61.13
56.20
55.93
55.60
55.25
26.39
160 150 140 130 120 110 100 90 80 70 60 50 40 30 20 ppm

Corydaline (**9**)

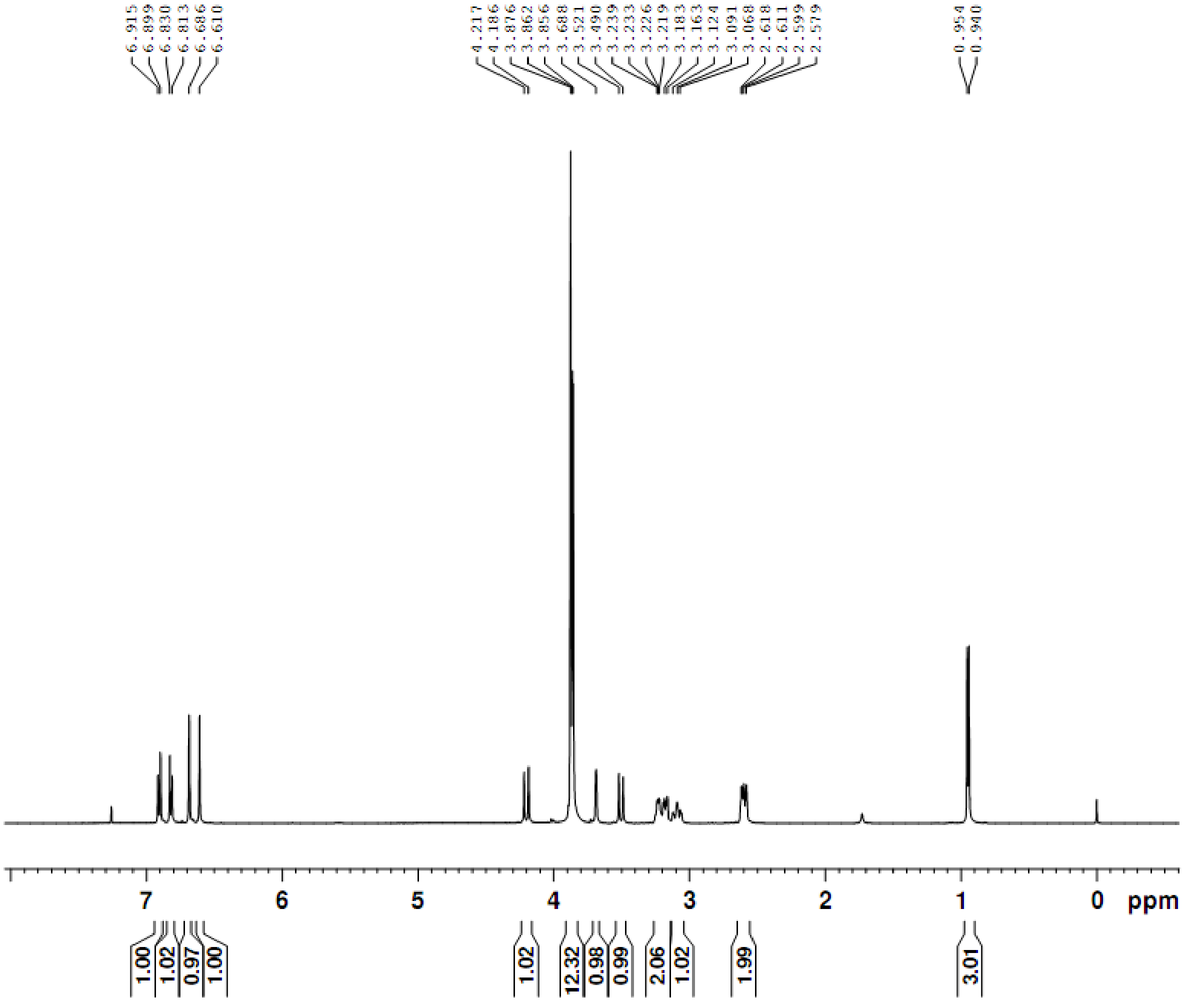

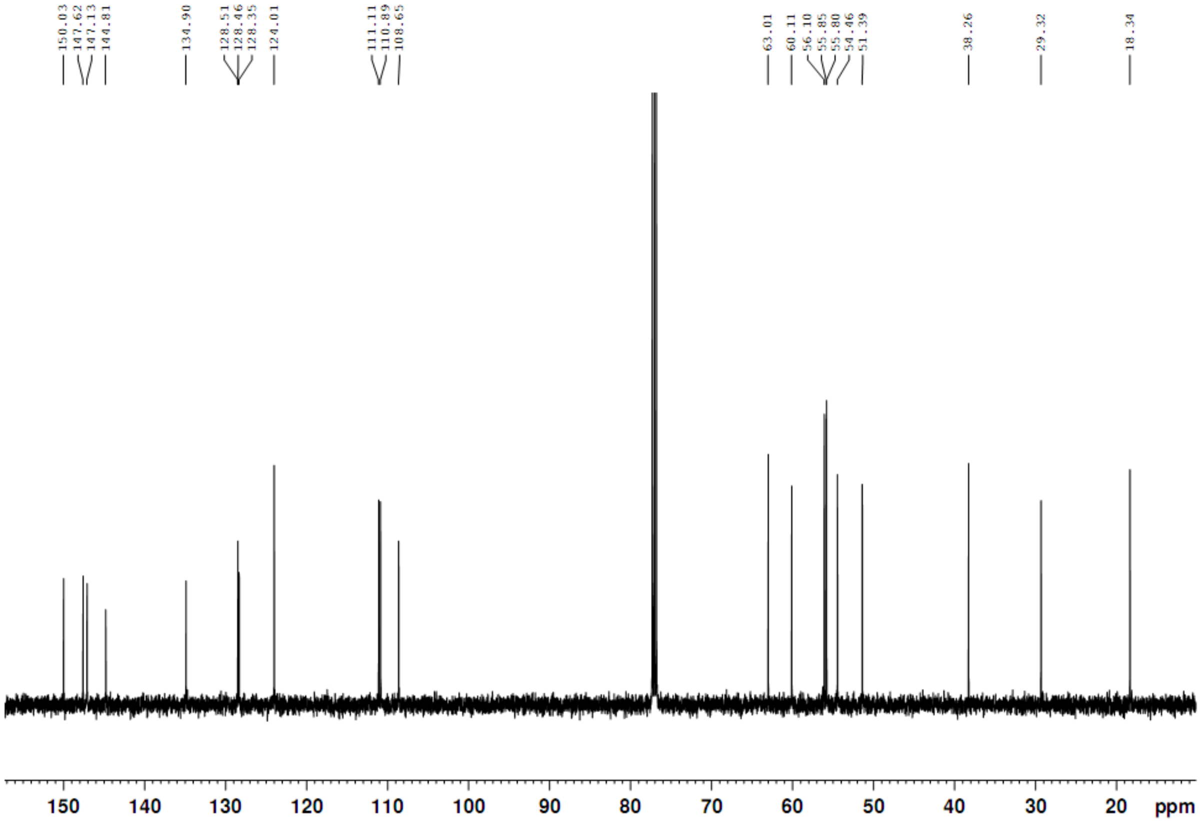

Dehydrocorydaline (**10**)

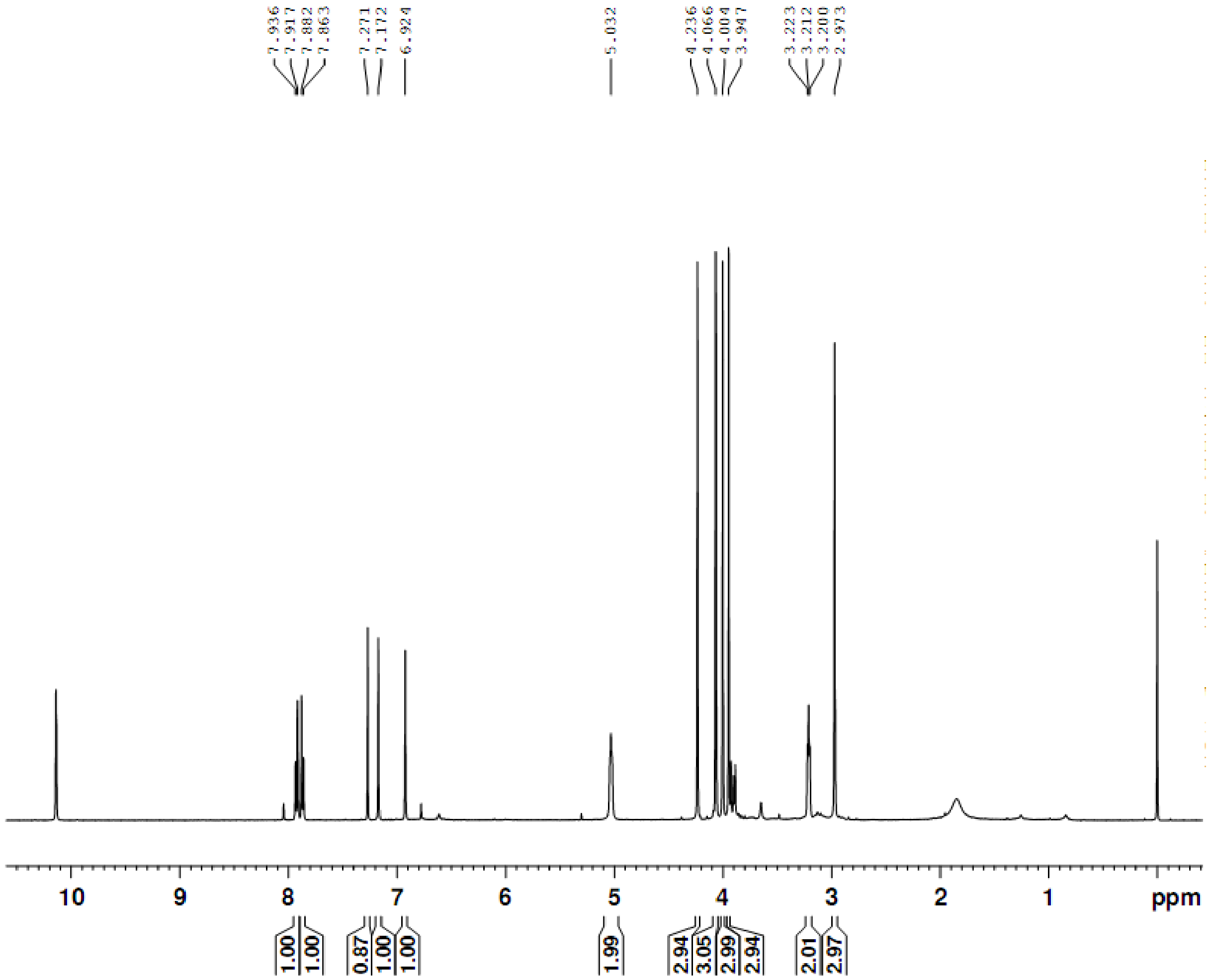

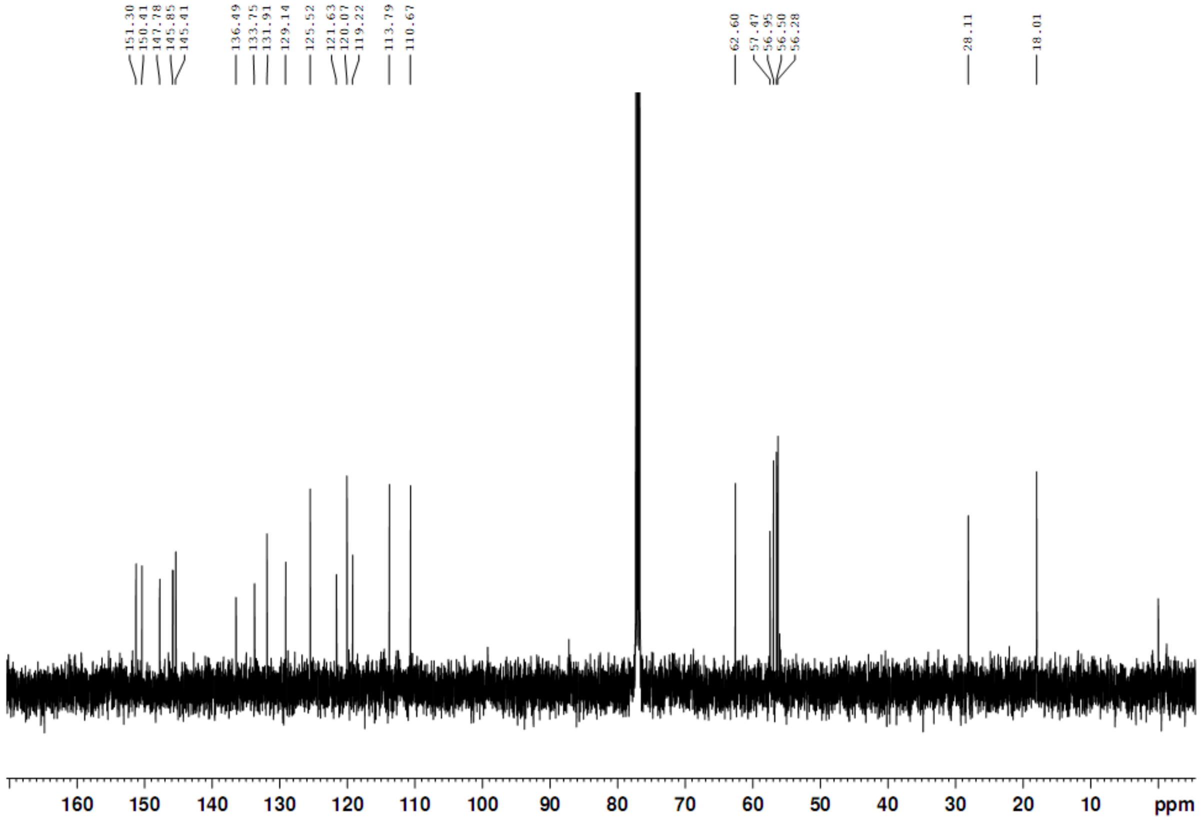

Supplement: Supplementary file 1 [file molecules-15-03556-s001.pdf]
